# Xeroderma Pigmentosum with Severe Neurological Manifestations/De Sanctis–Cacchione Syndrome and a Novel XPC Mutation

**DOI:** 10.1155/2017/7162737

**Published:** 2017-02-01

**Authors:** Esteban Uribe-Bojanini, Sara Hernandez-Quiceno, Alicia María Cock-Rada

**Affiliations:** ^1^Departamento de Dermatología, Universidad CES, Calle 10 A 22–04, Medellín, Colombia; ^2^Departamento de Endocrinología Pediátrica, Universidad de Antioquia, Carrera 51D 62-29, Medellín, Colombia; ^3^Unidad de Genetica Médica, Facultad de Medicina, Universidad de Antioquia, Carrera 51D 62-29, Medellín, Colombia

## Abstract

Several genetic disorders caused by defective nucleotide excision repair that affect the skin and the nervous system have been described, including Xeroderma Pigmentosum (XP), De Sanctis–Cacchione syndrome (DSC), Cockayne syndrome, and Trichothiodystrophy. Cutaneous photosensitivity with an increased risk of skin malignancy is a common feature of these disorders, but clinical manifestations commonly overlap these syndromes. Several genes have been found to be altered in these pathologies, but we lack more genotype-phenotype correlations in order to make an accurate diagnosis. Very few cases of DSC syndrome have been reported in the literature. We present a case of a 12-year-old Colombian male, with multiple skin lesions in sun-exposed areas from the age of 3 months and a history of 15 skin cancers. He also displayed severe neurologic abnormalities (intellectual disability, ataxia, altered speech, and hyperreflexia), short stature, and microcephaly, which are features associated with DSC. Genetic testing revealed a novel germline mutation in the XP-C gene (c.547A>T). This is the first case of an XP-C mutation causing De Sanctis–Cacchione syndrome. Multigene panel testing is becoming more widely available and accessible in the clinical setting and will help rapidly unveil the molecular etiology of these rare genetic disorders.

## 1. Introduction

Xeroderma Pigmentosum (XP) is a rare autosomal recessive disorder, first described by Hebra and Kaposi in 1874, which is caused by a defective nucleotide excision repair (NER) system, which produces mainly skin, ocular, and neurologic alterations [1, 2]. This abnormality in DNA repair can lead to XP, De Sanctis–Cacchione syndrome (DSC), Cockayne syndrome (CS), XP/CS complex, and Trichothiodystrophy or overlapping manifestations [3]. XP has been diagnosed worldwide and affects both genders equally. It is rare in Europe and North America with an incidence of 1 in 250.000–1.000.000 [4, 5] but is more frequent in areas with higher levels of consanguinity, like Japan (incidence of 1 in 20,000) Northern Africa (1 in 10,000–30,000) India, and the Middle East [6, 7].

**Figure 1:**
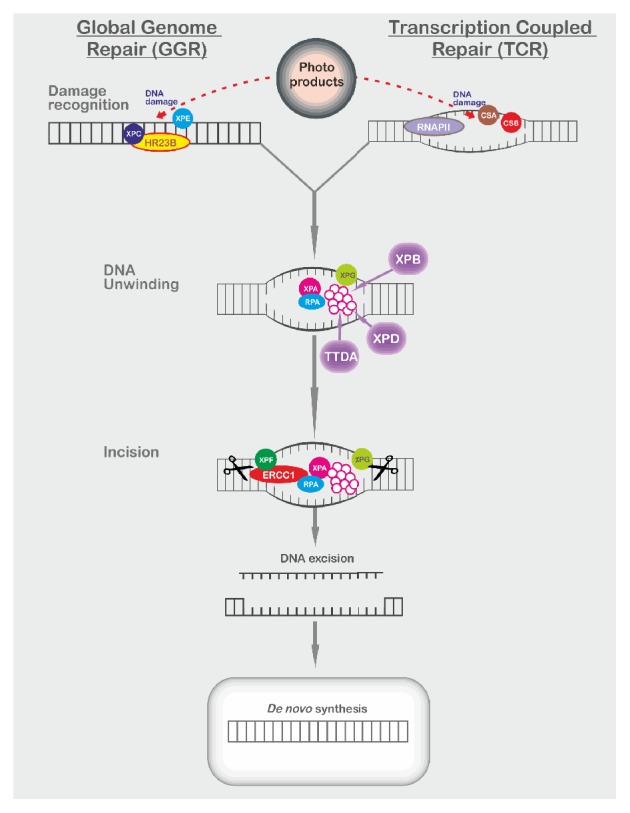
Nucleotide excision repair (NER) pathway. Transcription coupled repair (TCR) removes DNA damage from actively transcribed genes and global genome repair (GGR) from the rest of the genome [13]. TCR starts when RNA polymerase stops at the damaged DNA, which acts as a signal for CSA and CSB binding. In GGR, the XPE protein and the XPC-HR23B complex recognize the DNA damage. Upon initial recognition of the DNA lesion, both pathways converge. The XP-B and XP-D DNA helicases unwind the region surrounding the damaged site, along with the XP-A, XP-G, and replication protein A (RPA) [14]. The XPF-ERCC1 nuclease complex and XP-G endonuclease excise the damaged DNA. The resulting gap is replaced by de novo DNA synthesis.

XP is a genetically heterogeneous disease, which can be caused by pathogenic mutations in several genes involved in the NER pathway including the seven complementation groups XP-A to XP-G, and XP variant that is caused by deficient translesion synthesis. NER is responsible for repairing ultraviolet-induced photoproducts inside DNA and if a mutation is present in any of the components of the pathway, then the entire pathway fails to function normally (Figure 1) [8–10].

XP patients can be categorized into the different complementation groups, according to the mutated gene. The XPC group is the most common in the United States and Europe and is found in 43% of XP patients [11, 12].

Clinical characteristics vary depending on the complementation group, the nature of the mutation, and sun exposure, as well as other unknown factors. Some are common to all XP groups; approximately 50% of XP patients have acute sunburn reaction to minimal sun exposure. All patients have numerous lentigines (freckle-like hyperpigmented macules) on UV-exposed skin [15]. Symptoms usually start between 1 and 2 years of age. Sun exposure causes the skin to become dry with an increase in pigmentation and multiple premalignant actinic keratoses may develop at early ages. XP patients have a 10,000-fold increase in the risk of developing skin basal cell carcinoma and squamous cell carcinoma and a 2,000-fold increased risk of melanoma [16–18]. The median age of non-melanoma skin cancer in XP is 8 years, which is approximately 50 years earlier compared to healthy adults in the United States [2]. XP is also associated with a 20–50-fold increase in risk of internal malignancies, such as oral cavity, breast, uterine, brain, renal, gastric, testicular, and lung tumors and leukemia [19–21]. Ocular symptoms are common but primarily affect the anterior UV-exposed area, causing photophobia, conjunctival injection, and reduction in tears. Sun exposure leads to keratitis and corneal opacities and might cause ocular squamous cell carcinoma or melanoma [22]. Approximately 30% of XP patients present neurologic alterations, which may appear early in infancy or later in the second or third decade of life. They range from mild to severe, with intellectual disability, deafness, spasticity, and seizures [23]. The most severe form of neurologic alterations is known as* De Sanctis–Cacchione* syndrome: including classical XP manifestations plus hyporeflexia or areflexia, microcephaly, low IQ, progressive mental deterioration, athetosis, ataxia, spasticity, dwarfism, and hypogonadism [24, 25]. Shortening of the Achilles tendon may lead to quadriparesis [3]. Autopsies of XP patients have revealed loss of neurons mainly in the cerebellum and cerebrum. Neurologic alterations have been mainly observed in the group XP-A, XP-B, XP-D, or XP-G [12, 26]. Cockayne syndrome (CS) and Trichothiodystrophy (TTD) are disorders that have multisystem affection, with a wide range of symptoms and severity (Table 1) [27]. Patients with CS have a typical face phenotype (microcephaly, wizened face, deep-set eyes, mandible prognathism, hypoplastic teeth, and malformed ears) and develop dwarfism, hypogonadism, and neurological abnormalities such as progressive impairment of vision, hearing and speech, behavioral changes, intellectual disability, and problems with gait, leading to severe disability [3, 5]. TTD patients present hair and nails with sulphur deficiency, ichthyosis, and neurological affection [28]. Some reports have shown a combined DNA repair disorder, resulting in the XP/CS complex. This combination has been found in the complementation groups XP-B, XP-D, and XP-G, and only about 12 patients have been reported with XP/CS [29].

Although XP can be suspected by clinical features such as extreme UV sensibility in exposed areas and the appearance of numerous lentigines or skin cancer at early ages, molecular genetic testing is recommended for confirmation of the patient's mutation and complementation group characterization. This is important for genetic counseling, antenatal diagnosis, discussion of etiology, probability of occurrence in future generations, and the increased likelihood of occurrence in communities with high consanguinity.

XP has no cure, but skin complications can be controlled with adequate protection and prevention (sunblock, lip balm, covering clothing, UV-absorbing glasses, or even a facemask) [15]. Indoor environments should have UV film protection on windows, and fluorescent or halogen lights must be avoided. Premalignant lesions such as actinic keratosis should be treated with liquid nitrogen, topical imiquimod, or 5-fluorouracil [31, 32]. Oral isotretinoin has been effective in preventing the appearance of new neoplasms [33]. Topical DenV T4 endonuclease, a bacterial DNA repair enzyme, has been shown to reduce the appearance of new actinic keratoses in XP patients [34]. A routine evaluation by an ophthalmologist and a skin physician should be performed every 3–6 months depending on the severity of the disease. Vitamin D supplementation might be required due to deficiency from rigorous sun protection. Psychological advisory and support groups are very important for patients and their families. The management of patients with neurological abnormalities includes the use of hearing aid devices, with physical, occupational, and speech therapy [14]. Genetic counseling should be offered for families at risk. Antenatal diagnosis is possible in some countries by amniocentesis or chorionic villi sampling.

## 2. Case Presentation

**Figure 2:**
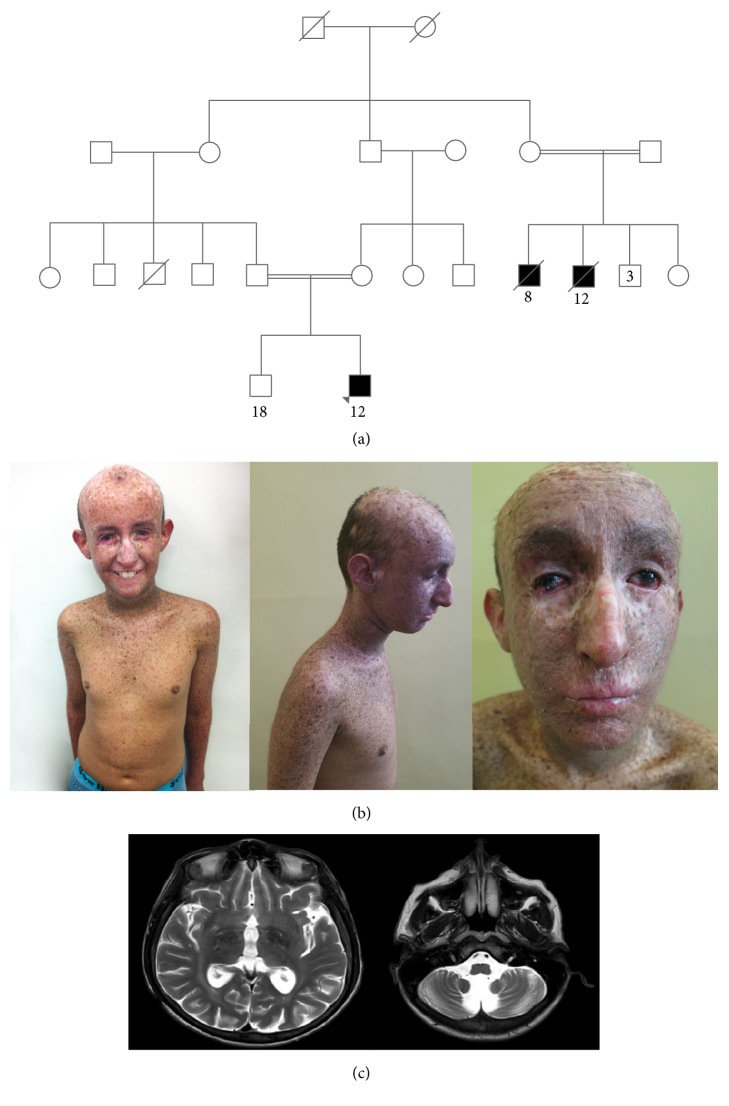
(a) Pedigree chart of the patient. The arrow indicates the index patient. Filled squares indicate affected individuals. (b) Photographs of the patient showing alopecia, freckle-like skin lesions, and actinic keratosis in sun-exposed areas. (c) Cerebral MRI showing parenchymal volume loss, cerebellar atrophy, and white matter gliosis.

We present the case of a 12-year-old Colombian male patient, son of first cousins, who was diagnosed with intrauterine growth restriction at 6 months prenatally and was born at term, with normal weight (3000 Kg) and height (49 cm) for gestational age (40 weeks). Since the age of 3 months he started developing multiple skin lesions, initially in the face, which then extended to the whole body, mainly in sun-exposed areas. The family history revealed similar recurrent skin cancers in 2 cousins on the maternal side of the family, who died at ages 8 and 12 years (Figure 2(a)). At the time of evaluation the patient had developed approximately 15 basal and squamous cell carcinomas in the face, ears, nose, eyes, and the extremities, which were surgically removed and no melanoma had been diagnosed. In addition to the dermatologic manifestations, the patient had been diagnosed with vitamin D deficiency due to excessive sun protection and also with detrusor hyperactivity plus detrusor-sphincter dyssynergia. Since infancy the patient also developed cognitive alterations, attention deficit/hyperactivity disorder, pes cavus, and progressive gait alterations. At 7 years he was diagnosed with bilateral cryptorchidism and hypogonadism by pediatric endocrinology. The physical exam revealed short stature (height: 133 cm) (<2 SD), microcephaly (head circumference: 47.5 cm) (<2 SD), low weight (26 Kg), intellectual disability, altered speech, ataxic gait, and hyperreflexia, accompanying the skin findings: alopecia, freckle-like skin lesions, actinic keratoses sparing unexposed areas, and marked skin atrophy in eyelids and around the mouth (Figure 2(b)). Electromyography of the extremities and auditory evoked potentials were normal. Spine MRI revealed a normal spinal cord and cerebral MRI showed parenchymal volume loss, cerebellar atrophy, and white matter gliosis (Figure 2(c)). A commercial Xeroderma Pigmentosum Next Generation Sequencing panel including the genes DDB2, ERCC2, ERCC3, ERCC4, ERCC5, POLH, XP-A, and XPC revealed a homozygous germline sequence variant designated c.547A>T in the XPC gene, which was confirmed by Sanger sequencing. This variant is predicted to result in premature protein termination (p.Lys183^*∗*^) and has not been previously reported in ClinVar and in The Human Gene Mutation Database, but is expected to be pathogenic. The karyotype was normal: 46XY with no structural or numerical chromosomal alterations. Genetic counseling, before and after genetic testing, was performed.

## 3. Discussion

We present a 12-year-old male with classical clinical manifestations of Xeroderma Pigmentosum and severe neurologic abnormalities (intellectual disability, ataxia, altered speech, and hyperreflexia), who carries a homozygous germline mutation in the XPC gene. Only 2 cases of XP with neurologic alterations have been described in the XPC complementation group; one was a 17-year-old female patient diagnosed with XPC and systemic lupus erythematosus, who presented with neurological and developmental abnormalities including microcephaly, intellectual disability, growth retardation, and primary amenorrhea [35]. The other was a 4-year-old patient from Korea with XPC who had hyperactivity, autistic features, and low levels of glycine [36].

Our patient's cerebral MRI revealed parenchymal volume loss, cerebellar atrophy, and white matter gliosis, which are characteristic of the De Sanctis–Cacchione syndrome (DSC). Even though XP with neurologic manifestations is still known sometimes as DSC, the latter is characterized by dwarfism and gonadal hypoplasia. Our patient shows classical features of DSC (intellectual disability, microcephaly, ataxia, hypogonadism, and dwarfism) with very early onset of skin lesions (3 months). Underlying molecular defects causing this disorder have been found in the ERCC6 (CS-B) gene, which encodes a DNA-binding protein that is important in transcription coupled excision repair and is also involved in Cockayne syndrome type B, as well as in XP-A and XP-D complementation groups [37, 38]. This would be the first report of an XP-C mutation in De Sanctis–Cacchione syndrome.

A differential diagnosis in this patient is XP/CS complex, which presents as XP and the presence of Cockayne syndrome features. The patient has some CS signs (hyperreflexia, dwarfism, microcephaly, and hypogonadism) but does not have other XP/CS complex characteristics such as intracranial calcification, normal-pressure hydrocephalus, deafness, spasticity, and pigmentary degeneration of the retina [15, 30]. Since the underlying molecular defect in XP/CS has been observed only in XP-B, XP-D, and XP-G, this would also be the first case of XP-C linked to XP/CS complex.

DSC and XP/CS complex have several overlapping clinical characteristics, making the distinction between both syndromes difficult. Even the same mutation in the CS-B gene has been associated with both CS and DSC phenotypes, indicating that sometimes there is no clear genotype-phenotype correlation and that other genetic or environmental factors could determine the phenotype [37]. An intriguing feature observed in this patient is the severe alopecia, which is unusual in classical XP or in any of the XP-associated disorders (Figure 2(b)).

There are very few cases of De Sanctis–Cacchione reported in the literature and the complete spectrum of the De Sanctis–Cacchione syndrome has been recognized in very few individuals. Most of the reported patients have been diagnosed based only on clinical findings. In some cases in vitro functional assays to screen for abnormalities in DNA repair of UV-irradiated cells have been performed. Cell-fusion techniques followed by assessment of DNA repair are also used to determine the complementation groups. However, the exact underlying genetic defect has not been explored in most reported cases. With the advent of new sequencing technologies, where multigene panel testing has become cheaper, faster, and therefore more accessible in the clinical practice, it is now possible to do genetic testing for multiple genes simultaneously. Since many patients have overlapping features of different syndromes caused by nucleotide excision repair defects, it is now possible to establish better genotype-phenotype correlations in these genetic disorders, according to the affected gene and the specific mutation found within each gene. In our patient, full sequencing of the coding regions of the genes XP-A, XP-B/ERCC3, XPC, XP-D/ERCC2, XPE/DDB2, XPF/ERCC4, XP-G/ERCC5, and XP variant/POLH and confirmation of variants by Sanger sequencing were performed, revealing a germline mutation in the XPC gene predicted to result in premature protein termination (p.Lys183^*∗*^). The majority of reported XPC mutations result in Premature Termination Codons (PTCs) [36, 39–42]. There are few reports of missense type mutations in the XPC gene [41, 43–45].

Genetic counseling is very important when managing mendelian disorders, especially in populations where consanguinity is very common. The patient's parents were first cousins and he had two affected cousins who had died at young age, who were also born to consanguineous parents. Therefore, it is important that the patient's parents and other relatives understand the risk of passing on an autosomic recessive disorder to their children. Once a genetic defect is found in a family, the carrier status can be assessed in other family members.

## 4. Conclusion

Defects in the nucleotide excision repair (NER) system cause rare entities including Xeroderma Pigmentosum, De Sanctis–Cacchione, Cockayne syndrome, and Trichothiodystrophy, which have higher incidence in areas with high levels of consanguinity. Since some of these syndromes have overlapping clinical features, it can be difficult in some instances to establish an accurate diagnosis. Molecular testing is necessary for a better classification of these disorders. The availability of faster and cheaper genetic testing methods will rapidly unveil the real genetic landscape underlying these disorders and a better genotype-phenotype correlation will allow us to manage better these patients. Direct mutation analysis can also be offered to at-risk individuals when a mutation is identified within a family. Although XP-mutation heterozygous carriers are clinically normal, there is active research regarding an increased cancer risk in these individuals.

## Figures and Tables

**Table 1:** Comparison of clinical manifestations present in our patient and the syndromes XP and XP with neurological manifestations, De Sanctis–Cacchione syndrome, CS, and XP/CS complex [5, 15, 27, 30].

	Characteristics	Patient	XP	XP with neurologic changes	De Sanctis–Cacchione syndrome	CS	XP/CS complex
*System*							
Skin	Malignant and premalignant lesions	X	X	X	X		X
	Photosensitivity	X	X	X	X	X	X

Ocular	Pigmentary degeneration of retina					X	X

Growth	Dwarfism	X			X	X	X
	Hypogonadism				X	X	X

Neurological	Microcephaly	X			X	X	X
	Mental retardation	X		X	X	X	X
	Ataxia	X			X	X	X
	Deafness			X	X	X	X
	Spasticity			X		X	X
	Seizures			X		X	
	Hyporeflexia or areflexia			X	X		X
	Hyperreflexia	X				X	X
	Pes cavus	X					

Other	Speech disability	X		X		X	

*Radiology*	Cerebral atrophy	X		X	X		
	Basal ganglia calcification					X	X
	Normal-pressure hydrocephalus					X	X

*Inheritance*		AR	AR	AR	AR	AR	AR

*Molecular defect*		XP-C	XP-A, XP-B, XP-C, XP-D, XP-E, XP-F, XP-G, variant	XP-A, XP-B, XP-D, XP-F	XP-A, XP-D, CS-B	CS-A, CS-B	XP-B, XP-D, XP-G
